# Foraging under uniform risk from different types of predators

**DOI:** 10.1186/1472-6785-8-19

**Published:** 2008-12-10

**Authors:** T Liesenjohann, JA Eccard

**Affiliations:** 1 Dep. of Animal Behavior, University of Bielefeld, Morgenbreede 45, 33501 Bielefeld, Germany; 2 Dep. of Animal Ecology, Institut für Biochemie und Biologie der Universität Potsdam Maulbeerallee 2a, 14469 Potsdam, Germany

## Abstract

**Background:**

Many animals live in environments where different types of predators pose a permanent threat and call for predator specific strategies. When foraging, animals have to balance the competing needs of food and safety in order to survive. While animals sometimes can choose between microhabitats that differ in their risk of predation, many habitats are uniform in their risk distribution. So far, little is known about adaptive antipredator behavior under uniform risk. We simulated two predator types, avian and mammalian, each representing a spatially uniform risk in the artificial resource landscapes. Voles served as experimental foragers.

**Results:**

Animals were exposed to factorial combinations of weasel odour and ground cover to simulate avian and/or mammalian predation. We measured short and long term responses with video analysis and giving-up densities. The results show that previously experienced conditions cause delayed effects. After these effects ceased, the risks of both types of predation caused a reduction in food intake. Avian predation induced a concentration on a smaller number of feeding patches. While higher avian risk caused a delay in activity, the weasel odour shortened the latency until the voles started to be active.

**Conclusion:**

We show that the voles differed in risk types and adjusted their feeding strategies accordingly. Responses to avian and mammalian risk differed both in strength and time scales. Uniformity of risk resulted in a concentration of foraging investment and lower foraging efficiency.

## Background

Predation influences the ecology of a prey species by directly increasing mortality and by altering prey behaviour indirectly (for reviews, see [1-3]). Antipredator behaviour includes foraging decisions [4], microhabitat shifts [5,6], and activity shifts [7,8]. In all situations investigated, essential trade-offs exist between antipredator behaviour and other fundamental activities, like foraging and mating [8,9].

Depending on the type and persistence of risks, the behavioural responses to short and long pulses of risk vary and include shifts of feeding effort to either safe periods (the predation risk allocation hypothesis [9]) or safe habitats [10-12]. However, what if there are no safe times and places to forage? If risk increases suddenly, animals can reduce activity, which lowers the energetic reserves of the individual [13]; however, if high risk is frequent or persistent, animals still need to feed and cannot continually avoid the risky periods [9]. In these cases, they should either posses permanent defences (e.g. chemicals or physical adaptations) or show behavioural adaptations. Due to the huge range of rodent predators, physical adaptations might not be as promising as behavioural plasticity. Thus, the difference in antipredator behaviour between high and low risk situations should decrease if periods of high risk are persistent [9]. Instead of allocating their effort to safer times, the animals might react by applying alternative feeding strategies.

In studies with heterogeneously distributed predation risk, rodents have been found to trade food in favour of safety by using safer microhabitats, indicating that animals consider relative levels of risk [14,15]. However, little is known in cases where risk is distributed uniformly in space and time. For example, in non-fragmented environments, an assessment of avian predation might not be possible and must therefore be assumed to be omnipresent. Furthermore, prey can face predators of the same locomotory type and body size as themselves, resulting in a uniform distribution of predation risk with no place for the prey to hide. For rodents, the least weasel *Mustela nivalis *(Linnaeus, 1766) is one such example of an omnipresent predator [16,17]. Behavioural adaptations to these types of persistent risk uniformity are poorly studied. Our own studies on foraging in risk homogeneity have suggested that foragers concentrate their feeding effort in few locations and accept lower feeding efficiency ([18]; Eccard and Liesenjohann 2008, under review). We interpret this trade-off in the context of local foraging decisions [8,19] and extrapolate the sum of local foraging decisions to a landscape level. Brown [8] extended Charnov's marginal value theorem [19] to show that foragers balance harvest rates with metabolic costs, predation risk, and opportunity costs. These studies focus on local differences in predation risk by assuming that the metabolic and opportunity costs are constant. However, under uniform predation risks, the local predation risk in different food patches does not vary and the metabolic costs are also assumed to be constant among patches. Thus, foraging decisions depend on the costs of other activities besides foraging in a local patch. For example, the opportunity costs might be low under high uniform risk; since other activities and other animals underlie the same high risk as the forager, the foragers exploit patches to low quitting-harvest rates. In low risk situations, alternative opportunities like territory defence and mate search might gain importance because all animals underlie the same conditions and overall activity will be higher under low risk. Thus, the distribution of feeding effort over a greater area might be a consequence of high activity and engagement in multiple opportunities.

Even though most animals live in environments with multiple predators (e.g., hawks and owls, canids or snakes [20]), surprisingly few studies have assessed the conflicts arising from life under the threat of different types of predators ([21-24], but see Sih (1998) [24] for a review). Behavioural responses that reduce mortality from a predator type may expose the animal to greater mortality from a second predator type [19], so a predator-induced shift of habitat may send prey into "the jaws, talons or fangs of another species of predator" [22]. Small mammals face more than the problem of two predators in different microhabitats; they have to deal with different types of predation at the same time. Most studies dealing with more than one predator have regarded their effects as additive, which is difficult to address in a biological and statistical sense [24]. For example, the availability of cover [25] and scents of mammalian predators [26] heavily alter foraging behaviour [27] and can change with the season [28] or the time of a day [29,30]. How these factors interact when occurring simultaneously in uniform risk landscapes remains unstudied.

An individual has to decide when and where to forage and, in order to maximise fitness, has to take a given risk context into account. Animals respond adaptively to changes in predation risk and exhibit several strategies to minimise their exposure to predators [22,31,32]. Therefore, foraging decisions can be used as an indicator of how animals perceive their environment [8]. We investigated the foraging behaviour of small mammals in depletable food patches and simulated two types of uniform risk, avian and mustelid. Avian predation risk was manipulated by ground cover (first factor, either high or low), whereas mustelid predation risk was manipulated by the presence of an odour of a least weasel (*Mustela nivalis*) (second factor, odour present or not). Foragers were exposed to high or low avian and mammalian predation risk levels in a 2 × 2 factorial set up.

In high risk situations, animals should tend to avoid travelling between sources and value known food resources more than uncertain findings. Additionally, the option of fulfilling alternative tasks becomes less attractive because opportunities like mate search or territory defence are linked to a greater exposure to predators.

We predict that, under high, uniformly distributed risk, animals will concentrate their foraging effort to fewer trays and expect different temporal scales for the two risk types. Responses to mustelid predation are expected to be observed on a short time scale because predator odour is a reliable sign of predator presence [27]. The risk by avian predation is difficult to estimate for a ground dwelling rodent. Birds of prey can cover plains very quickly, and their presence or absence is almost unpredictable for the prey [31]. Therefore, we expect that, in higher avian risk treatments, the avian predation risk is perceived as a constant threat over the entire experimental time scale and should also lead to a concentration of effort. We aim to dissect short term and long term responses by the video analysis of short term responses, such as foraging delay and behaviour in the first foraging bout after application of odour treatments, medium term responses such as concentration of effort and number of bouts in the first six hours after a change of treatment, and long term changes by comparing the first and second day of a treatment. With the factorial set-up, we further aim to discover interactions among the two risk types.

## Methods

### Animals

Twelve wild caught male bank voles (*Myodes glareolus*, (Schreber, 1780)) served as experimental foragers. The animals were randomly assigned to three groups, because only four test arenas were available. Each animal of a group of four was tested for a total of 18 days. The first four individuals were tested in March, individuals 5–8 in April, and individuals 9–12 in May 2006. One was excluded during the experiment due to the absence of a response to any of the treatments. Animals were habituated to the experimental arenas and to the required mode of feeding from seed-trays for 4 days followed by an experiment where only ground cover was manipulated for six days (reported in [33]). After this experiment, all animals were kept under ground cover (wire mesh) for two days to provide similar starting conditions and independence from former experimental groups for the experiment reported in this paper. Animals were habituated indoors to a 12:12 hour L: D cycle, which was adjusted until darkness started at 12.00 to ensure activity during the treatment phases. Animals were weighed prior to each experiment, after the habituation phase, and after each experiment. All animals lost weight in the four-day habituation phase prior to experiment one, and their weights were constant for the duration of the experiment (mean initial body weight ± 1SE: 25,9 ± 3,3 gr., Mean weight loss: 2, 6 ± 1, 4 gr.). Consecutive groups contained breeding states from sexually inactive animals (March) to animals in an intermediate state in the second group (April), followed by fully sexually active animals (May). This difference among the groups was accounted for by including a three-level factor "season" into the statistical model.

### Arenas and treatments

Indoor arenas of 9 m^2 ^contained a 5 cm high layer of sand and were surrounded by 100 cm high steel walls. Each animal was provided with a shelter consisting of a small (12 × 7 cm, 4 cm high) box in the middle of the arena throughout the whole treatment sequence. Above each arena, infrared cameras were installed using a 50 W red-light bulb per arena.

We utilized a 2 × 2 factorial design examining the effects of predation (present or absent) crossed with ground cover (present or absent):

1. The presence or absence of predator odour was used to simulate predation risk by a terrestrial predator. Cage bedding of a least weasel (*Mustela nivalis*) was distributed before each observation period over all seed trays, making all feeding stations equally dangerous. The odour donor was a male least weasel fed a diet of *Microtus arvalis *and *Myodes glareolus*. Every fourth day, the cage bedding was removed and frozen at -20° until used in the experiment. As a control, we used clean, frozen cage bedding.

2. The presence or absence of ground cover, simulating low or high avian predation risk. For cover, we provided a wire mesh of 1 cm mesh size that was installed on small stilts 4 cm above the ground and through which we were able to video tape movements.

The four combinations of the two treatments were assigned to the 12 animals in 8 different orders, since the odorous treatments had to be applied simultaneously to all arenas within the research hall. In the first and third groups, combinations of odours were administered during the 2^nd ^and 4th treatment, and, in the second group, the odours were administered during the 1^st ^and 3^rd ^treatment. Treatment orders differed among animals in each season; i.e., the four different combination orders applied in the first season were repeated in the third season. Treatments started at 12:00 am and lasted for two days (46 h). During two observation periods (OP) animals very digitally video taped. The first OP started with the change of the treatment at 12.00 and lasted 6 hours, the second started 24 hours later and lasted again 6 hours. Variables from the videos of the OPs are later compared within and between treatments (see Statistics). Treatment conditions were changed on days 1, 3, 5, and 7 between 10–12:00 am. After the OPs, all seed trays were sifted and the cage bedding was removed if necessary.

### Foraging grids

Foraging decisions can be measured using artificial, non-refilling food patches with declining harvest rates over patch exploitation. Theory predicts that a forager will stop depleting a patch when the benefits of the harvest rate no longer justify energetic-, predation- and missed opportunity costs while foraging (based on the marginal value theorem [19,31,34]). In the patch, the giving-up density (GUD) of food relative to substrate can be measured [31].

During the observation periods, 25 feeding stations were evenly distributed in the arena in a 5 × 5 grid. The feeding stations were 5 cm high and were dug into the sand, with 50 cm between feeding stations. Each feeding station contained 400 ml of sand and 0.2 gr. of millet. Thus, a total amount of 5 grams of food was provided per observation period (6 hrs). Since the animals consumed a maximum of 30% of the food provided, different feeding strategies (either sampling a high number of trays with a high harvest rate or depleting a low number of trays to a low food level) were possible.

Between the observation periods, the animals were kept under treatment conditions with 10 food patches with even food distribution that were removed three hours before each observation period.

### Statistics

To analyse the delayed effects of the previous treatments on the intake and use of trays in the first and second observation periods (OPs) of the current treatment, we used a linear mixed model (lmm) and included the observation period (day, 2 levels) with a first order autoregressive co-variation model of repeated measurements. We used the second, third, and fourth treatment combinations and added the risk relation to the respective earlier treatment quality as a factor (higher, lower, or same risk). The dependent variables were intake and number of used trays in OP 1 (or OP 1). Higher risk was assumed if avian or mammalian risk levels increased while the other was constant or if both were increased; however, a lower risk was assumed if avian or mammalian risk levels were decreased while the other was constant or if the risk levels of both factors were decreased. The same risk level was assumed if the risk level of one factor increased and that of the other factor decreased. The current treatment was included into the analysis as a three level factor (high risk: both predation types present; medium: either avian or mammalian risk present; or low: no risk present).

Foraging decisions were identified with landscape-wide measures based on GUDs in trays over the entire OP. Decisions were analysed as follows: the total consumption was used as an indicator of perceived risk, the number of seed trays visited (as indicated by food prints) was used as a measure of tray exploration, and the concentration of effort (i.e., the percentage of food collected from the 5 most depleted trays) was used as a measure of the spatial concentration of effort. Behaviour was analysed using the video material. We used the number of activity bouts of the entire OP as a measure of overall activity. A bout was defined as an activity period outside the shelter which lasted at least 5 minutes and was divided from the next bout by 30 minutes of inactivity. Additionally, we analysed the number of trays used in the first bout and the delay of activity after onset of the experimental night as indicators of short term effects.

We analysed dependent variables with linear mixed models for the effects of odour treatment (2 levels), avian predation risk (2 levels), season (3 levels), and position of treatment in experimental sequence (4 levels) within individuals with the lmm procedure using SPSS 15 (SPSS Inc., Chicago, Illinois). Animals were used as subjects and treatment order as repeats. Response variables were log transformed to obtain homogeneous variances if necessary. If no homogeneous variances could be achieved, non-parametric Friedman and Wilcoxon tests were applied.

The main effects and the 2-way interactions of the fixed factors (odour, avian risk and season) were inspected in the first model. Non-significant interactions (p > 0.05) were subsequently removed until the most parsimonious models were obtained. The position of the treatment in the experimental sequence had no effect on any of the models and was removed from all reported models.

## Results

### Delayed effects as a result of predation risk allocation

In the first observation period (1st OP) of a treatment, food intake and the number of trays used were strongly influenced by the relative risk of the previous treatment and interaction between the current and previous treatments (Table 1). After the higher risk treatments, the voles consumed more food and used more trays than after the less dangerous treatments (Table 1). In the second OP, only the risk level of the current treatment explained the intake and the number of seed trays used.

**Table 1:** Delayed effects of previous treatments on current treatments

OP	Variable	Food intake (g)			Nr of used trays		
	Factor	df	F	p	df	F	p
1	Current treatment	2,24	1,11	0.35	2,27	0,2	0.84
	Previous treatment	2,24	2,75	0.084	2,27	3,98	0,03
	Curr. Treat.* Prev. treat.	2,18	4,31	0.03			

2	Current treatment	2,17	7,76	0.04	2,20	6,82	0.006
	Previous treatment	2,17	0,05	0.04	2,20	1,67	0.21
	Curr. Treat.* Prev. treat.	2,10	2,14	0.17			

The relative risk of the previous treatment (three risk levels: higher, lower, or same risk) compared to the present treatment (three risk levels: high, medium, or low) shows a significant impact on variables obtained after a change of treatment. During the first observation period (OP 1, 0–6 hours after change of treatment), the previous and current treatments interact significantly. In the second OP (24–30 hours after change of treatments), only the current treatment causes the significant effects. 11 bank voles served as experimental foragers.

To further analyse effects of persistent risk treatments only, we analysed the second observation period by itself.

### Behavioral changes during a treatment due to uniform risk distribution

Over all treatments, the animals decreased their food intake by one half when the animals were shifted from the safest treatment combination (low avian risk, no mustelid odour 0.67 g ± 0.45) to a combination with both risks (0.32 g ± 0.3). Food intake was reduced by both high avian and mustelid predation risk with no interaction (Table 2). Under the increased avian risk, food intake was reduced by 35 percent (low: 0.64 g ± 0.41, high: 0.44 g ± 0.31,) while the application of mustelid odour reduced food intake by 26 percent (without: 0.62 g ± 0.36, with: 0.46 g ± 0.36). Note: All values are means ± 1 SD.

**Table 2:** Behavioural responses of bank voles foraging in a risk-uniform landscape.

Variable	Intake (g)			Nr of Trays			Conc. Of Effort			Nr. of Bouts			Latency		
Factor	df	F	p	df	F	p	df	F	p	df	F	p	df	F	p
Weasel Odour	1,24	8,20	0.008	1,23	0,12	0.73	1,19	2,800	0.11	1,6	14,07	0.009	1,8	7,92	0.022

Av. Pred, Risk	1,30	1,40	0.001	1,31	0,19	0.008	1,32	5,500	0.026	1,10	7,180	0.022	1,11	6,11	0.031

Season	2,9	1,25	0.332	2,10	0.83	7,90	2,10	1,400	0.28	2,8	1,740	0.239	2,50	0,004	0.95

Avian Pred. * Seas.										2,10	8,930	0.002			

Weasel. Od. * Seas.										2,6	20,41	0.006			

Treatments were weasel odour (present or absent) and avian predation risk (ground cover present or absent) in an ongoing season (three months). The most parsimonious linear mixed models are shown. Non-significant factor interactions were removed.

**Figure 1:**
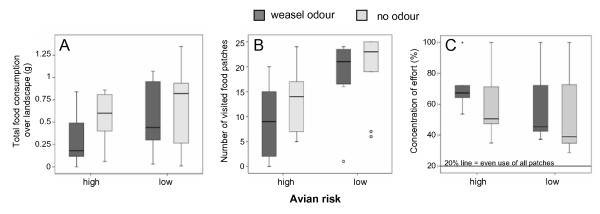
**A-C: Food consumption and distribution of feeding effort of 11 bank voles in a 6 hr long observation period, effects of higher or lower avian predation risk and weasel odour treatment**. A: Food intake (gram millet). B: The number of used trays C: Concentration of effort (percentage of food taken from the five most depleted trays (20% of trays)).

The number of trays used and the concentration of effort were only affected by higher avian predation risk but not by weasel predation risk (Table 2, Fig. 1B+C). With cover, 24 ± 8 trays were visited, while without cover, 11 ± 7 trays were visited. Even though the concentration of effort was highest in the treatment group with both risk factors present (72% ± 16 were foraged from the 5 most used trays), only avian predation increased the concentration significantly from a mean of 55% ± 23 with cover to a mean of 65% ± 19 without cover (Table 2).

**Figure 2:**
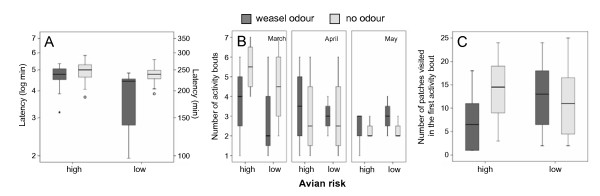
**A-C: Behavioural observations of foraging behaviour of 11 bank voles under avian and mammalian predation risk in three different months**. A: The delay of activity after the application of odour treatments. B: Number of bouts within a 6 hr observation period. C: Nr of visited trays in the first foraging bout.

During the first bout after the application of experimental conditions, animals used 11 ± 9 of the 25 trays, but the number of trays visited differed between the four treatment combinations within animals (Friedman test chi2 = 9.95, p = 0.019, Figure 2C). This difference was due to the reduced number of trays visited by the voles when they were exposed to the mustelid predation risk in combination with high avian predation risk (7 ± 6) when compared to the number of trays visited in the absence of mustelid predation risk in the presence of high avian predation risk (15 ± 7) (post hoc test (Wilcoxon) Z = 2.31, p = 0.021). The two other treatments showed no significant difference (all Z < 1.5, p > 0.1).

Video analysis revealed that the length of delay and the number of bouts were each dependent upon both the avian and weasel predation risks (Table 2, Figs. 2A+B). The highest number of bouts occurred when only avian predation risk existed (3.7 ± 2 bouts), whereas the lowest number of bouts occurred when only the weasel odour was present (2.9 ± 1.4 bouts). The longest delay until foraging occurred when no cover was provided and no weasel odour was presented (157 ± 89 minutes). Surprisingly, the shortest delay until foraging occurred when only the weasel odour was presented (82 ± 60 min). Furthermore, the number of bouts varied between the groups tested in different seasons. In the early season, a mean number of 4 ± 2 activity phases were counted. In the second season, 3.1 ± 1.7 phases were counted. In the third season, 2.5 ± 0.8 phases were counted (Table 2).

## Discussion

### Strategic foraging patterns in risk uniform environments

Foraging decisions under predation pressure have mainly been studied in settings where prey animals were offered a choice of feeding stations that differed in predation risk [14,15,35]. Prey usually shift foraging activity to safer habitats, but many natural habitats are risk uniform. Predation risk can be evenly spread across the habitat, such as when predator and prey are of similar body size and locomotion type [17] or the habitat is structurally uniform.

We created a risk uniform habitat in experimental arenas and showed that foragers changed their feeding strategy by concentrating their effort on fewer trays under increased uniform risk. Since trays were not refilled over the observational period, foragers yielded and accepted diminishing returns over time. Consequently, foragers under uniform predation risk are less efficient. This trade-off can be interpreted as balancing the harvest rates to the predation risks and opportunity costs [8]. In a uniform high predation risk, the opportunity costs of other activities other than foraging in a local patch are low since other activities and other animals underlie the same high risk. Therefore, animals exploit patches to low quitting-harvest rates, while dangerous travelling is avoided.

### Temporal allocation

The effects of the previous treatments dominated the effects of the current treatments during the first observation period (OP) if the previous treatment posed a higher risk (Table 1). This effect indicates that the animals compensate by increasing their feeding rate and supports the predictions of the predation risk allocation hypothesis [9]. According to Lima and Bednekoff [9], the duration of low and high risk periods should affect the amount of antipredator behaviour allocated to the respective periods, and, at some point, animals have to counterbalance for earlier missed feeding opportunities. If animals avoid foraging during periods of high risk, their energetic resources decrease if high risk situations become persistent, and their nutritional demands will surpass their fear of predation. Voles, just as any other homeothermic small animal, can not interrupt their food intake too long [36] because they need to maintain a favourable energy balance. In our experiment, voles adjusted their feeding strategies to the persistent risk level and predation type only after a day had lapsed. Thus, in constantly shifting environments, special behavioural adaptations can be explained by the influences of previous conditions. If animals start their activities with a memory of the prior state of the foraging and risk landscape, their decision making might be based on three different factors: their own condition (depending on the former activities), their memory of the last environmental state, and updates of current changes in the actual state of environment and food resources, representing a Bayesian forager [37,38] that is regulated by its internal state of nutrition.

### Specific responses to different types of predation

In the second observation period, the avian predation risk level had already persisted for at least 24 hours. Weasel odour was applied at the start of each observation period, and it affected short term behaviour and reduced the number of visited trays in the first bout. However, over all bouts in the six hours, the difference was no longer visible.

Uniform avian predation risk significantly reduced the food intake of the voles (Table 2, Fig. 1A), increased their concentration of effort (Fig. 1C), increased their delay in activity (Fig. 2A), and reduced the number of used feeding stations (Fig. 1B) when compared to the same animals under lower uniform risk levels.

The importance of avian predation risk is that it is the most invariant result in studies of risk heterogeneity [8,31,39]. Birds of prey have large home ranges and scan the ground out of sight of their prey. As such, voles have to treat avian predation risk as a constant. The prey seem to be unable to adjust feeding activities to the proximate cues of avian presence, but they are able to adjust to the overall perception of the environmental quality in terms of cover and refuge. This is supported by the long delay of activity in the avian risk treatment groups.

The responses of voles to cues that indicate the presence of a weasel have been demonstrated earlier, including spacing behaviour [40] and feeding behaviour [41]. The least weasel is about the same size as its prey, is able to move as voles do [16], and follows voles into their burrows [17], which cause a uniform distribution of weasel predation risk. Weasel presence also alters the temporal patterns of vole activity [14]. Support for weasel odour effects in our experiment comes from the strong reduction of overall food intake (Fig. 1A) with the weasel treatment. On a short time scale during the first of all activity bouts after the distribution of fresh weasel odour, voles reduced their explorative activities if avian predation risk was high at the same time, as indicated by the number of used trays (Fig. 1B). A short delay until the start of activity and general boldness were also observed in fish that were raised under conditions with a predator threat, as compared to those fish raised without the presence of a threat [42]. This may be interpreted as a sign of alertness under constant predation pressure.

### Interactions

Our 2 × 2 factorial set-up allowed us to check for (emergent) multiple predator effects (MPE [24]). According to Lima [43], the addition of predation risk to an already risky situation (no cover and weasel odour in our case) should not cause prey to use time for predator scanning but rather to shift to a concentration of foraging investment. Supporting this model, the foragers in our study reduced the number of foraging patches in reaction to simulated mustelid risk only if avian predation risk was already high, this can be seen as an emergent effect which only occurs when two types of predation occur simultaneous. Additionally, animals reduced their food intake under persistent risk of avian predation and the renewed risk of mammalian predation. These responses lead to a minimum intake when both predation types are present, whereas each of the predation types alone produces a similar reduction of intake (Fig. 1A, boxes 2 and 3). Although we found additive effects of the two predation types in the food intake, the combination of both predation types did not differ from the sum of their single effects (no emergent effects) in this variable. A further reduction of time spent foraging (indicated by a delay of activity) allowed the animals to reduce metabolic needs. Thus, two or more types of predation (representing "trophic species" [24]) can produce additive and emerging effects, depending on the type of risk that is represented through these predators.

### Effects of season

Our experiments were conducted over the progression of spring. Even though animals were kept under artificial light conditions, we cannot exclude effects of an internal clock of the animals [44] and their change of sexual status. These factors could have changed the relative importance of parameters like mate search or territory defence, which could explain the significant interaction with season (Fig. 2B).

## Conclusion

Our study has shown that voles adapt their feeding strategies to the perceived levels of uniform risk and that their feeding strategies are specific for different classes of predation. They changed feeding strategies according to the type of risk on spatial and temporal scales. Additive and emergent effects of the two risk types were found, but no multiplicative effects. This might be due to the small margins and energy spares that small mammals have, which do not allow additional reduction of activity at a certain benchmark. The animals adapted their spatial distribution of feeding effort to the uniform distribution of risk. With all places being equally unsafe, animals concentrated their effort to very few trays. Thus, foragers were able to perceive risk homogeneity over the entire landscape and adapt foraging strategies to reduce their risk. Because voles are capable of adaptive strategies to risk uniformity, it stands to reason that voles experience such landscapes of fear under natural conditions and have evolved optimal feeding strategies to deal with permanent uniform risk distributions.

## Authors' contributions

TL participated in design of the study, collected data, performed parts of the statistical analysis and wrote and drafted the manuscript. JAE conceived the study, participated in the design of the study, and performed parts of the statistical analysis. She revised the manuscript and added important intellectual content.
